# Supplementary material for: Automatic Segmentation of Retinal Fluid and Photoreceptor Layer from Optical Coherence Tomography Images of Diabetic Macular Edema Patients Using Deep Learning and Associations with Visual Acuity
Source: Biomedicines. 2022 May 29;10(6):1269. doi: 10.3390/biomedicines10061269 (PMC9220118; doi:10.3390/biomedicines10061269)
Supplement: Supplementary file 1 [file biomedicines-10-01269-s001.zip › Supplementary Table S1.pdf]

Supplementary Table S1. Layers in different modules in EfficientNet-B5 [30]

	**Module 1**	**Module 2**	**Module 3**	**Module 4**	**Module 5**
Layer1	Depthwise Convolution	Convolution	Global Average Pooling	Multiply	Multiply
Layer2	Batch Normalization	Batch Normalization	Rescaling	Convolution	Convolution
Layer3	Activation	Activation	Convolution	Batch Normalization	Batch Normalization
Layer4	X	X	Convolution	X	Dropout
