# Supplementary figures and images for: Automatic Segmentation of Retinal Fluid and Photoreceptor Layer from Optical Coherence Tomography Images of Diabetic Macular Edema Patients Using Deep Learning and Associations with Visual Acuity
Source: Biomedicines. 2022 May 29;10(6):1269. doi: 10.3390/biomedicines10061269 (PMC9220118; doi:10.3390/biomedicines10061269)

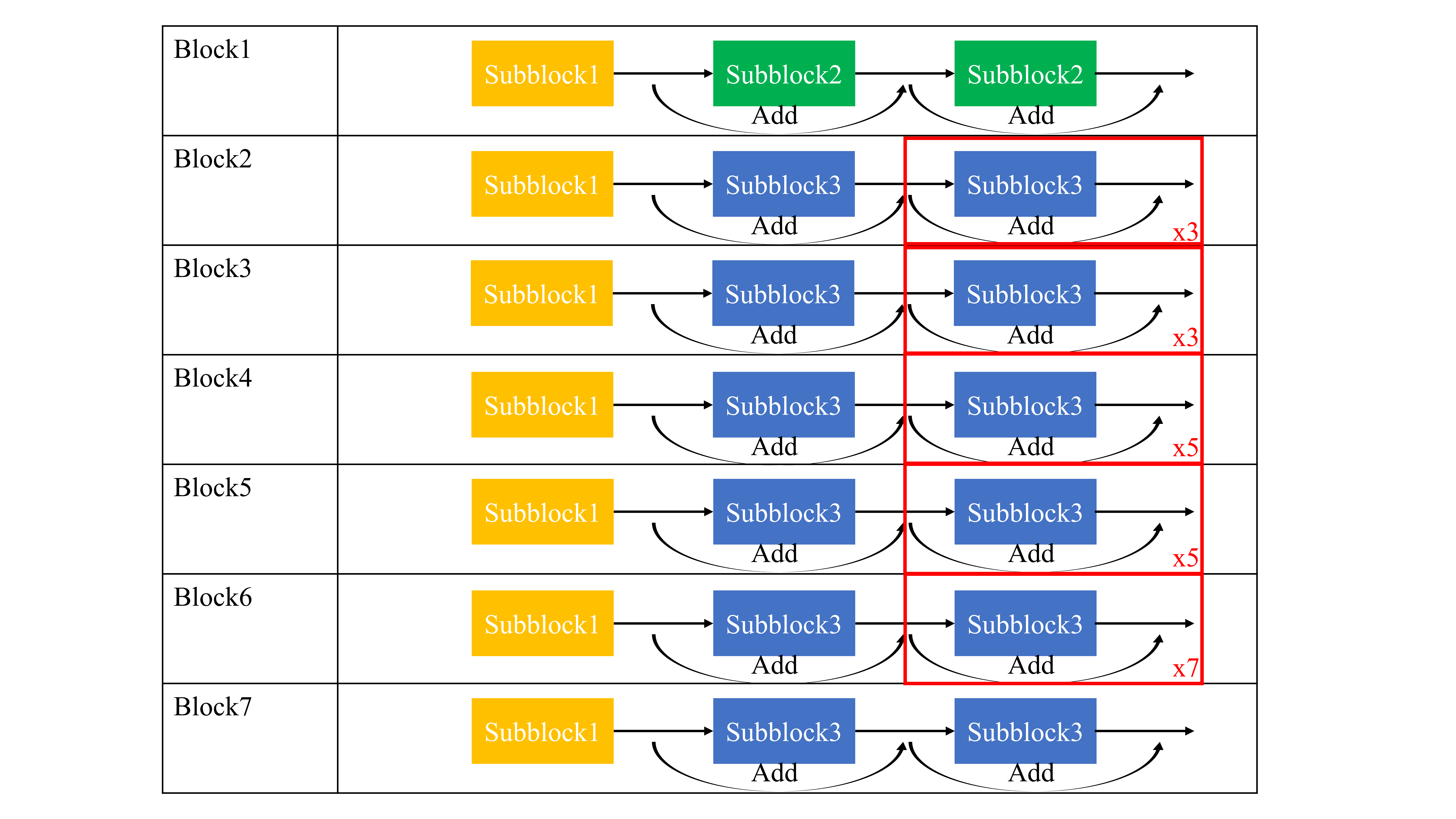

Supplement: Supplementary file 1 [file biomedicines-10-01269-s001.zip › supplementary Figure S1.tif]

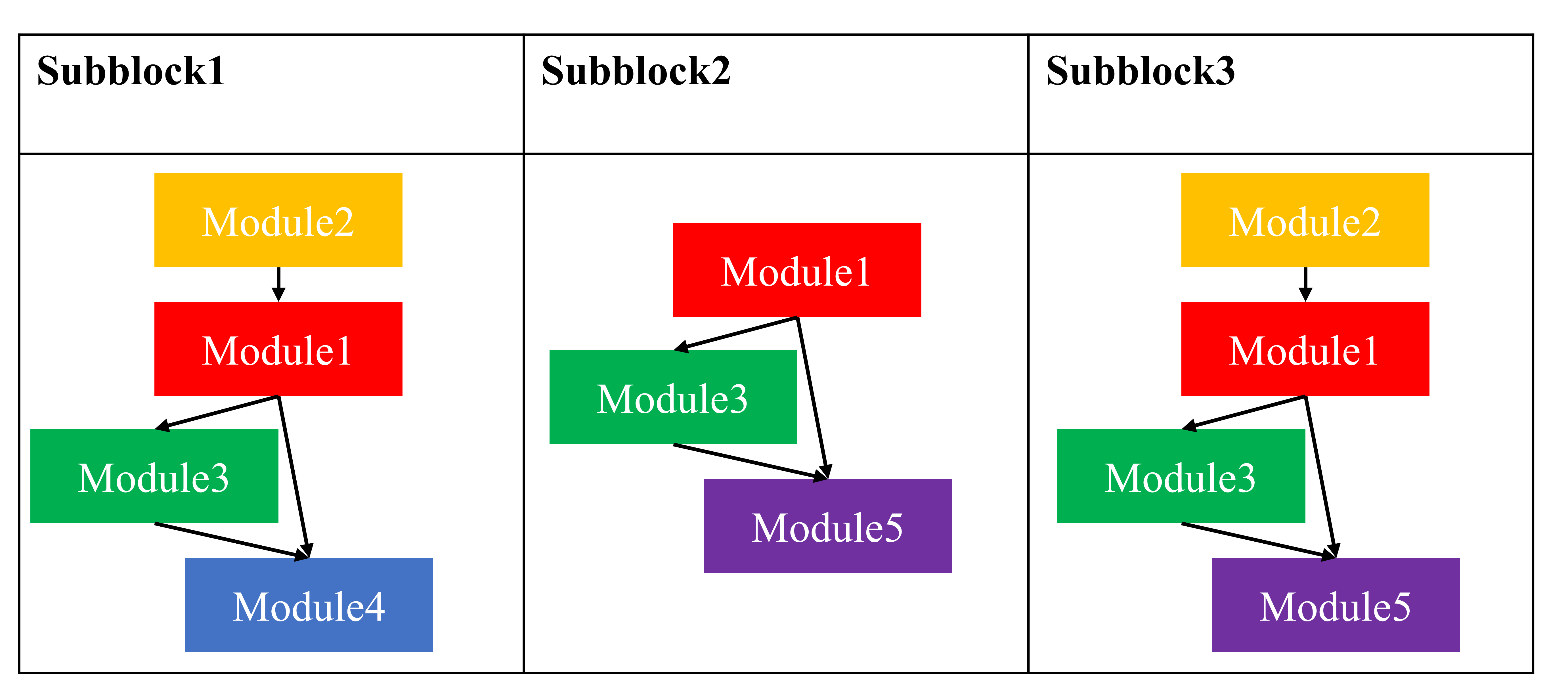

Supplement: Supplementary file 1 [file biomedicines-10-01269-s001.zip › supplementary Figure S2.tif]
